# IMPACTITIS: THE IMPACT FACTOR MYTH SYNDROME

**DOI:** 10.4103/0019-5154.48998

**Published:** 2009

**Authors:** Mohamed L Elsaie, Jenna Kammer

**Affiliations:** *From the Department of Dermatology and Cutaneous Surgery, University of Miami, FL, USA*; 1 *From the Drexel University School of Medicine, Philadelphia, PA, USA*

**Keywords:** *Impact Factor*, *Journal Citation Report*, *Science Citation Index*

## Abstract

**Background:**

In the early 1960s, Eugene Garfield and Irving Sher created the journal impact factor to help select journals for the *Science Citation Index (SCI).* Today it has become a widespread subject of controversy even for Garfield, the man who created it who is quoted saying “*Impact Factor is not a perfect tool to measure the quality of articles but there is nothing better and it has the advantage of already being in existence and is, therefore, a good technique for scientific evaluation*”. The use of the term “impact factor” has gradually evolved, especially in Europe, to include both journal and author impact. This ambiguity often causes problems. It is one thing to use impact factors to compare journals and quite another to use them to compare authors. Journal impact factors generally involve relatively large populations of articles and citations. Individual authors, on average, produce much smaller numbers of articles.

**Objectives:**

Impact factor, an index based on the frequency with which a journal's articles are cited in scientific publications, is a putative marker of journal quality. However, empiric studies on impact factor's validity as an indicator of quality are lacking. The authors try to evaluate and highlight the validity of Impact Factors and its significance as a tool of assessment for scientific publications.

**Methods:**

Analysis of the several reports in literature and from their own point of view.

**Conclusion:**

A journal's impact factor is based on 2 elements: the numerator, which is the number of citations in the current year to any items published in a journal in the previous 2 years, and the denominator, which is the number of substantive articles (source items) published in the same 2 years. The impact factor could just as easily be based on the previous year's articles alone, which would give an even greater weight to rapidly changing fields.

## Introduction

Being the most popular dermatologist does not mean that you are the most highly valued and considered the most prestigious one. Similarly, being a high selling writer making the *New York Times* best sellers' 10 list, does not mean that you are being tipped as a favorite for the Nobel Prize in literature. Similarly, making it to MTV's top 10 pop chart for a year doesn't crown one the king of pop!!!

The impact factor is only one of three standardized measures created by the *Institute of Scientific Information (ISI)*, which can be used to measure the way a journal receives citations to its articles, over time. Citations to articles published in a given year rise sharply to a peak between two and six years after publication. From this peak, citations declines over time. The citation curve of any journal can be described by the relative size of the curve (in terms of area under the line), the extent to which the peak of the curve is close to the origin, and the rate of decline of the curve. These characteristics form the basis of the ISI indicators impact factor, immediacy index and cited half-life. The impact factor is a measure of the relative size of the citation curve in years 2 and 3. It is calculated by dividing the number of current citations a journal receives to articles published in the two previous years by the number of articles published in those same years. So, for example, the 1999 Impact factor is the citations in 1999 to articles published in 1997 and 1998 divided by the number articles published in 1997 and 1998. The number that results can be thought of as the average number of citations the average article receives per annum in the two years after the publication year.

The ISI in Philadelphia serves as a continuous record of scientific citations. The references are rearranged to show how many times each publication has been cited within a certain period of time and by whom, and the results are published as the scientific citation index (SCI). On the basis of the *Science Citation Index* and authors' publication lists, the annual citation rate of papers by a scientific author or research group can thus be calculated. Similarly, the citation rate of a scientific journal - known as the journal impact factor - can be calculated as the mean citation rate of all the articles contained in the journal.[1] Journal impact factors, which are published annually in *SCI Journal Citation Reports (JCR)*, are widely regarded as a quality ranking for journals and used extensively by leading journals in their advertising. A list of the JCR ranking of some dermatology journals in 2005 is shown in [Table 1].

Apart from being non-representative, the journal impact factor is encumbered with several shortcomings of a technical and more fundamental nature. The factor is generally defined as the recorded number of citations within a certain year (for example, 1996) to the items published in the journal during the two preceding years (1995 and 1994), divided by the number of such items (this would be the equivalent of the average citation rate of an item during the first and second calendar year after the year of publication). However, the Science Citation Index database includes only normal articles, notes, and reviews in the denominator as citable items, but records citations to all types of documents (editorials, letters, meeting abstracts, etc) in the numerator; citations to translated journal versions are even listed twice.[2,3] Because of this flawed computation, a journal that includes meeting reports, interesting editorials, and a lively correspondence section can have its impact factor greatly inflated relative to journals that lack such items. Editors who want to raise the impact of their journals should make frequent reference to their previous editorials, since the database makes no correction for self citations. The inclusion of review articles, which generally receive many more citations than ordinary articles,[4,5] is also recommended. Furthermore, because citation rate is roughly proportional to the length of the article,[6] journals might wish to publish long, rather than short, articles. Dynamic research fields with high activity and short publication lags, such as biochemistry and molecular biology, have a correspondingly high proportion of citations to recent publications - and hence higher journal impact factors - than, for example, ecology and mathematics.[7]

It is widely assumed that publication in a high impact journal will enhance the impact of an article (the ‘free ride’ hypothesis). In a comparison of two groups of scientific authors with similar journal preference, who differed twofold in mean citation rate for articles, the relative difference was the same (twofold) throughout a range of journals with impact factors of 0.5 to 8.0.[8] If the high impact journals had contributed ‘free’ citations, independently of the article contents, the relative difference would have been expected to diminish as a function of increasing journal impact.[9] These data suggest that the journals do not offer any free ride. The citation rates of the articles determine the journal impact factor (a truism illustrated by the good correlation between aggregate citation rates of article and aggregate journal impact found in these data), but not vice versa.

It is still believed the journal's impact factor determines the prestige of the published article but that is unfortunately true and it is the article quality that should determine the journal's prestige. In some institutes and organizations worldwide, the IF is used as a premise for the evaluation of an individual's resume and professional qualities for allocation of a position or a post, often for promotion from one post to another among university professionals. This comes at the expense of the quality or value of the work and its scientific impact rather than the journal's impact.[10,11] Some of the problems encountered with using IF as a way of evaluating journal standings are highlighted in [Table 2].

**Table 1:** Journal Citation Report 2005 for top 5 dermatology journals

Abbreviated journal title	ISSN	IF	Total cites	Articles
J Invest Dermatol	0022-202x	4.406	17500	318
Arch Dermatol	0003-987x	3.434	11321	192
Brit J Dermatol	0007-0963	2.978	13597	365
Contact Dermatitis	0105-1873	2.701	3967	103
J Am Acad Dermatol	0190-9622	2.402	14528	383

**Table 2:** Major disadvantages of using an IF as the sole method of assessment

Cons of relying on an impact factor for evaluation
Journal impact factors are not statistically representative of individual journal articles Journal impact factors correlate poorly with actual citations of individual articles Review articles are heavily cited and inflate the impact factor of journals Long articles collect many citations and give high journal impact factors Short publication lag allows many short term journal self citations and gives a high journal impact factor Selective journal self citation: articles tend to preferentially cite other articles in the same journal Books are not included in the database as a source for citations Database has an English language bias Database is dominated by American publications Impact factor is a function of the number of references per article in the research field Research fields with literature that rapidly becomes obsolete are favored Impact factor depends on dynamics (expansion or contraction) of the research field Small research fields tend to lack journals with high impact Citation rate of an article determines the journal impact, but not vice versa Availability of research material to scientists and researchers worldwide determine their pattern of citation i.e. some references are not available for many scientists worldwide Language knowledge affects the number of articles cited for a publication

## Conclusion

For evaluation of scientific quality, there seems to be no alternative to qualified experts reading the publications. Much can be done, however, to improve and standardize the principles, procedures, and criteria used in evaluation, and the scientific community would be well served if efforts could be concentrated on this rather than on developing ever more sophisticated versions of basically useless indicators.
